# Surgeon experience in hip arthroscopy improves operative efficiency and reduces conversion to total hip arthroplasty: A meta‐analysis

**DOI:** 10.1002/ksa.70431

**Published:** 2026-06-26

**Authors:** Nikolai Ramadanov, Maximilian Heinz, Robert Hable, Roland Becker, Ingo J. Banke

**Affiliations:** ^1^ Center of Orthopaedics and Traumatology, Brandenburg Medical School University Hospital Brandenburg an der Havel Brandenburg an der Havel Germany; ^2^ Faculty of Health Science Brandenburg Brandenburg Medical School Theodor Fontane Brandenburg an der Havel Germany; ^3^ Faculty of Applied Computer Science Deggendorf Institute of Technology Deggendorf Germany; ^4^ Clinic of Orthopaedics and Sports Orthopaedics, School of Medicine and Health TUM University Hospital, Technical University of Munich Munich Germany; ^5^ AGA‐Society for Arthroscopy and Joint‐Surgery, Hip Committee, c/o Walder Wyss Ltd. Zurich Switzerland

**Keywords:** hip arthroscopy, learning curve, meta‐analysis, surgeon experience

## Abstract

**Purpose:**

Hip arthroscopy (HAS) is technically demanding, and surgeon's experience may influence outcomes. However, the magnitude and domain‐specific effects of the learning curve remain unclear.

**Methods:**

A systematic review and meta‐analysis were conducted according to Preferred Reporting Items for Systematic Reviews and Meta‐Analyses (PRISMA) and registered in PROSPERO (CRD420251185937). PubMed/MEDLINE, Embase and Scopus were searched through 31 December 2025 for studies comparing early versus late experience phases in HAS. Random‐effects meta‐analyses (Sidik–Jonkman estimator with Hartung–Knapp adjustment) were performed for operative and traction time, patient‐reported outcomes, complications, revision arthroscopy and conversion to total hip arthroplasty (THA). Risk of bias was assessed using ROBINS‐I and certainty of evidence using Grading of Recommendations, Assessment, Development and Evaluation (GRADE).

**Results:**

Eighteen studies (2624 procedures; 1291 early and 1333 late cases) were included. Late cases showed significantly shorter operative time (−31.77 min; 95% confidence interval [CI] −51.40 to −12.14) and traction time (−15.67 min; 95% CI −25.42 to −5.92), with substantial heterogeneity. Functional outcomes favoured late experience, including higher improvement in minimal clinically important difference units (0.58; 95% CI 0.17 to 0.99) and modest gains in modified Harris Hip Score (2.78 points; 95% CI 0.16 to 5.41) and Non‐Arthritic Hip Score (8.41 points; 95% CI 1.99 to 14.83). Complication rates and revision arthroscopy did not differ significantly, whereas conversion to THA was lower in late cases (odds ratio [OR] 0.10; 95% CI 0.01 to 0.98). Most studies were at moderate to serious risk of bias, and certainty of evidence was low to very low.

**Conclusion:**

Surgeon experience in HAS is associated with improved operative efficiency and modest functional gains, while safety outcomes remain largely unchanged. The lower THA conversion rate in late cases may reflect improved patient selection and decision‐making, although confidence is limited by study design and heterogeneity.

**Level of Evidence:**

Level III systematic review and meta‐analysis of predominantly retrospective cohort studies.

## Graphical abstract

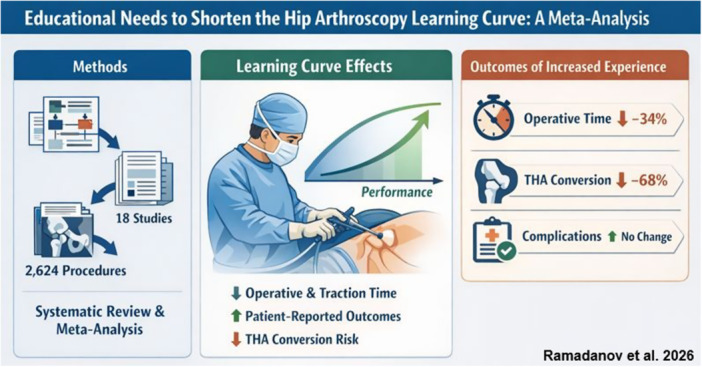

**Abbreviations**
CI: confidence interval
GRADE: Grading of Recommendations, Assessment, Development and Evaluation
HHS: Harris Hip Score
HOOS: Hip Disability and Osteoarthritis Outcome Score
HOS‐SSS: Hip Outcome Score—Sports‐Specific Subscale
*I* ^2^
: inconsistency index
MCID: minimal clinically important difference
MD: mean difference
mHHS: modified Harris Hip Score
NAHS: Non‐Arthritic Hip Score
OR: odds ratio
PRISMA: Preferred Reporting Items for Systematic Reviews and Meta‐Analyses
PROM: patient‐reported outcome measure
ROBINS‐I: Risk Of Bias In Non‐randomized Studies—of Interventions
SD: standard deviation
THA: total hip arthroplasty

## INTRODUCTION

Hip arthroscopy (HAS) has become a cornerstone procedure for diagnosing and managing intra‐articular hip disorders such as femoroacetabular impingement syndrome (FAIS), labral tears, and chondral lesions. With expanding indications and increasing procedural complexity, surgeon experience and the associated learning curve have emerged as key determinants of safety and outcome.

Across orthopaedic surgery, procedural mastery follows a measurable trajectory. A recent systematic review of learning curves in sports orthopaedics reported substantial variability in case numbers required for competence, ranging from 22 for shoulder arthroscopy to >70 for hip procedures, underscoring the technical demands of HAS [31]. In HAS specifically, prior reviews have demonstrated reductions in complication rates, operative and traction times, and revision rates with increasing experience, while patient‐reported outcomes remained favourable [9, 14]. However, these studies primarily described trends qualitatively without providing pooled estimates across key domains [9, 14, 31]. Consequently, the magnitude and clinical relevance of experience‐related differences remain unclear. Recent evidence from hip preservation surgery suggests that learning‐curve effects may primarily influence operative efficiency, while safety and radiological outcomes remain relatively stable, although data remain limited [27].

Simulator‐based training may improve technical performance, although transferability to operative proficiency remains uncertain [8]. While overall complication rates in HAS are low, outcomes remain dependent on surgeon experience [10]. Despite these insights, the quantitative characteristics of the HAS learning curve—its magnitude and domain‐specific effects—are insufficiently defined and likely vary depending on procedure type and context.

Therefore, the present systematic review and meta‐analysis aims to quantify the learning curve in HAS by comparing early versus late experience phases. Specifically, this study aims to (1) determine the magnitude of improvement across operative and clinical outcomes, (2) identify domains most affected by experience and (3) provide an evidence‐based framework for training and quality assurance in HAS. We hypothesized that increasing surgeon experience would be associated with improved operative efficiency and functional outcomes, while complication rates would remain unchanged.

## METHODS

### Protocol and registration

This systematic review and meta‐analysis were conducted in accordance with Preferred Reporting Items for Systematic Reviews and Meta‐Analyses (PRISMA) guidelines [23] and prospectively registered in PROSPERO (CRD420251185937). The PRISMA checklist is provided in the Supporting Information (Table S1).

### Eligibility criteria

Eligible studies included patients undergoing HAS for intra‐articular pathologies (e.g., FAIS, labral tears, chondral lesions), comparing different stages of surgeon experience (e.g., chronological grouping or cumulative sum [CUSUM]‐defined early vs. late phases), and reporting at least one relevant outcome (operative or traction time, complications, revision, total hip arthroplasty [THA] conversion or patient‐reported outcome measures [PROMs]). Prospective or retrospective cohort studies, registry studies and case series (>20 procedures) were included. Technical notes, reviews, case reports, cadaveric or simulation studies and studies without clear experience stratification were excluded.

### Search strategy

PubMed/MEDLINE, Embase and Scopus were searched from inception to 31 December 2025 using terms related to HAS and learning curves. Reference lists of included studies and relevant reviews were screened. Two reviewers independently performed study selection, with disagreements resolved by consensus or a third reviewer.

### Study selection

Records were managed in Rayyan for duplicate removal and blinded screening. Two reviewers independently assessed titles, abstracts and full texts against predefined criteria.

### Data extraction

Data were extracted using a standardized, piloted form. Extracted variables included study characteristics, cohort size, patient demographics, definition of the learning curve and outcome data for early versus late phases. When multiple temporal groups were reported, early and late phases were selected or pooled to enable direct comparison. Graphical data were extracted using established methods [12, 29], and authors were contacted if necessary.

### Minimal clinically important difference (MCID)‐based outcome normalization

PROMs were harmonized using MCID‐based normalization as previously described [26], enabling pooling across instruments on a unified scale.

### Quality assessment

Risk of bias was assessed using Risk Of Bias In Non‐randomized Studies—of Interventions (ROBINS‐I) [34], and certainty of evidence using Grading of Recommendations, Assessment, Development and Evaluation (GRADE) [11]. Publication bias was evaluated using funnel plots and Egger's test [6]. Sensitivity analyses excluded outliers and high‐risk studies.

### Data synthesis and statistical analysis

Random‐effects meta‐analyses (Sidik–Jonkman estimator with Hartung–Knapp adjustment) were performed when ≥3 studies reported comparable outcomes [28]. Continuous outcomes were analysed as mean differences (MDs) and dichotomous outcomes as odds ratios (ORs), both with 95% confidence intervals. Heterogeneity was assessed using *I* ^2^, *τ* ^2^ and Cochran's *Q*.

## RESULTS

### Systematic literature search

**Figure 1:**
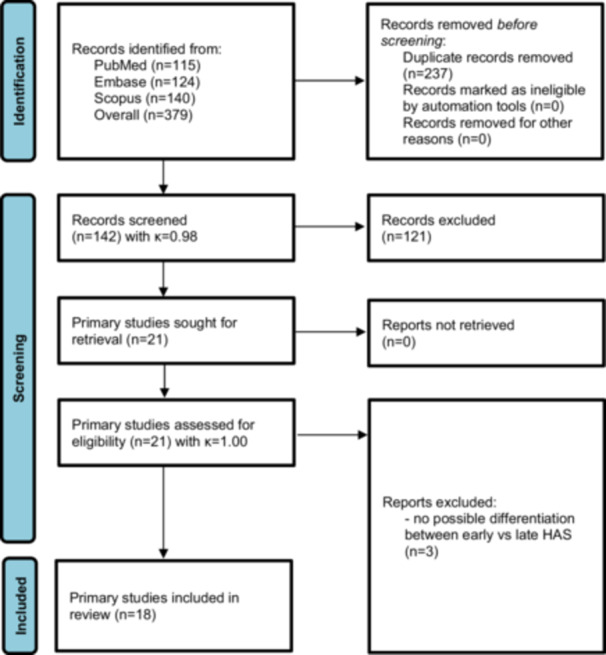
PRISMA flow diagram of study selection. Flow diagram illustrating the identification, screening, eligibility assessment and inclusion of studies. A total of 379 records were identified, 237 duplicates were removed, and 21 full‐text articles were assessed for eligibility. Ultimately, 18 primary studies met all criteria and were included in the quantitative synthesis. HAS, hip arthroscopy; PRISMA, Preferred Reporting Items for Systematic Reviews and Meta‐Analyses.

The database search identified 379 records (PubMed 115, Embase 124 and Scopus 140). After removal of duplicates (*n* = 237), 21 full‐text articles [2, 3, 4, 5, 7, 13, 15, 16, 17, 18, 19, 20, 21, 24, 25, 30, 32, 33, 36, 37, 39] were assessed, with excellent inter‐reviewer agreement (*κ* = 0.98–1.0). Three studies were excluded due to unclear stratification of experience phases [13, 20, 33]. Ultimately, 18 studies [2, 3, 4, 5, 7, 15, 16, 17, 18, 19, 21, 24, 25, 30, 32, 36, 37, 39] were included (Figure 1). Outcome harmonization was applied in studies with multiple phases [2, 5, 15, 17, 32] and graphical data extraction [36, 39].

### Descriptive patient characteristics

A total of 2624 procedures (1291 early; 1333 late) were analysed (Table 1). Sample sizes ranged from 40 to 360. Age and sex distributions were generally comparable between early and late cohorts. Mean age ranged from approximately 27 to 45 years, and the proportion of male patients ranged from 29% to 66%. BMI was less consistently reported but showed similar distributions between groups when available (approximately 22.6–29.0 kg/m^2^).

**Table 1:** Characteristics of included primary studies.

First author	Origin	Journal (ISSN)	Study design	Level of evidence	Early cases, *N*	Late cases, *N*	Age, years ± SD (range)	Male sex, *N* (%)	BMI, kg/m^2^ ± SD (range)	Follow‐up period (months)
Boden et al. 2014 [2]	United Kingdom	*Acta Orthopaedica Belgica* (ISSN 0001‐6462)	Prospective cohort study	IV	40	40	39 (14–67)	NR	NR	23 (12–43)
Dietrich et al. 2014 [3]	Germany	*Knee Surgery, Sports Traumatology, Arthroscopy* (ISSN 0942‐2056)	Retrospective comparative cohort study	III	85	86	NR	NR	NR	NR
Domb et al. 2020 [4]	United States	*Arthroscopy – The Journal of Arthroscopic and Related Surgery* (ISSN 0749‐8063)	Retrospective comparative cohort study	III	200	200	Early: 36.7 ± 13.9 Late: 37.2 ± 15.6	Early: 63/187 (33.7%) Late: 55/189 (29.1%)	Early: 25.3 ± 4.8 Late: 25.9 ± 5.2	≥24
Dumont et al. 2020 [5]	United States	*Arthroscopy – The Journal of Arthroscopic and Related Surgery* (ISSN 0749‐8063)	Retrospective cohort study	IV	75	75	NR	NR	NR	NR
Flores et al. 2018 [7]	United States	*The Orthopaedic Journal of Sports Medicine* (ISSN 2325‐9671)	Prospective cohort study	II	30	30	Early: 37.2 ± 11.5 Late: 35.3 ± 10.8	Early: 15/30 (50%) Late: 17/30 (56.7%)	Early: 25.6 ± 4.0 Late: 25.1 ± 4.5	
Kautzner et al. 2017 [15]	Czech Republic	*International Orthopaedics (SICOT)* (ISSN 0341‐2695)	Prospective cohort study	II	50	50	37 (16–69)	64/150 (42.7%)	NR	24
Kern MJ et al. 2018 [16]	United States	*Journal of the American Academy of Orthopaedic Surgeons* (ISSN 1067‐151X)	Prospective case series	IV	50	50	30.1 ± 11.8 (13–62)	37/100 (37%)	25.1 ± 4.8	≤9
Konan et al. 2011 [17]	United Kingdom	*The Journal of Bone and Joint Surgery – American Volume* (ISSN 0021‐9355)	Prospective case series	IV	30	30	32 (19–55)	39/100 (39%)	29 (21–39)	≥24
Kuschnaroff Contreras et al. 2010 [18]	Brazil	*Revista Brasileira de Ortopedia* (ISSN 0102‐3616)	Retrospective case series	IV	75	75	37.3 (12–58)	69/150 (46%)	NR	≥24
Lee et al. 2013 [19]	South Korea	*Knee Surgery, Sports Traumatology, Arthroscopy* (ISSN 0942‐2056)	Retrospective case series	IV	20	20	Early: 40.9 ± 17.1 Late: 42.0 ± 15.3	Early: 12/20 (60%) Late: 8/20 (40%)	Early: 23.0 ± 2.7 Late: 23.4 ± 2.9	6
Nossa et al. 2014 [21]	Colombia	*Current Orthopaedic Practice* (ISSN 1940‐7041)	Prospective cohort study	II	150	210	40.4 ± 12.0 (15–79)	147/362 (40.6%)	NR	≥6
Park et al. 2014 [24]	South Korea	*Arthroscopy – The Journal of Arthroscopic and Related Surgery* (ISSN 0749‐8063)	Retrospective case series	IV	100	97	44.6 (19–70)	97/200 (48.5%)	NR	28.2 (19–42)
Philippon et al. 2020 [25]	United States	*Knee Surgery, Sports Traumatology, Arthroscopy* (ISSN 0942‐2056)	Retrospective cohort study	IV	100	100	Early: 37.1 ± 12 Late: 33.5 ± 12	Early: 62/100 (62%) Late: 56/100 (56%)	NR	≥24
Schüttler KF et al. 2018 [30]	Germany	*Archives of Orthopaedic and Trauma Surgery* (ISSN 0936‐8051)	Retrospective cohort study	III	60	60	43.9	254/529 (48.0%)	26.9	≥1.5
Smith KM et al. 2017 [32]	United States	*Arthroscopy – The Journal of Arthroscopic and Related Surgery* (ISSN 0749‐8063)	Retrospective case series	IV	50	50	33.0 ± 11.5	48/100 (48%)	26.0 ± 4.2	NR
Tassinari E et al. 2025 [36]	Italy	*Journal of Experimental Orthopaedics* (ISSN 2198‐1964)	Retrospective case series	IV	36	35	NR	42/71 (59.2%)	22.6 ± 2.2 (18.7–26.5)	90 ± 22 (60–132)
Vilchez F et al. 2010 [37]	Mexico/Spain	*Acta Ortopédica Mexicana* (ISSN 2306‐4102)	Prospective cohort study	V	30	67	Early: 27 (25–52) Late: 29 (15–51)	Early: 19/30 (63%) Late: 44/67 (66%)	NR	≥6
You JS et al. 2020 [39]	United States	*Orthopaedic Journal of Sports Medicine* (ISSN 2325‐9671)	Prospective cohort study	II	110	58	35.3 ± 9.6	77/168 (46%)	25.07 ± 3.98	24

Abbreviations: BMI, body mass index; NR, not reported; SD, standard deviation.

### Risk of bias

Most studies were rated at moderate to serious risk of bias, primarily due to confounding and participant selection. Classification of interventions was generally low risk, while missing data, outcome measurement and reporting were mostly moderate. No study was rated as low risk across all domains (Table 2).

**Table 2:** Risk of bias assessment using the ROBINS‐I tool.

Study	D1 Confounding	D2 Selection	D3 Classification	D4 Deviations	D5 Missing data	D6 Outcome measurement	D7 Reporting bias	Overall
Boden et al. [2]	Serious	Moderate	Low	Low	Moderate	Moderate	Moderate	Serious
Dietrich et al. [3]	Serious	Serious	Low	Low	Moderate	Moderate	Moderate	Serious
Domb et al. 2020 [4]	Moderate	Moderate	Low	Low	Low	Moderate	Moderate	Moderate
Dumont et al. 2020 [5]	Serious	Moderate	Low	Low	Low	Moderate	Moderate	Serious
Flores et al. 2018 [7]	Moderate	Moderate	Low	Low	Low	Low	Moderate	Moderate
Kautzner et al. 2017 [15]	Moderate	Moderate	Low	Low	Low	Low	Moderate	Moderate
Kern et al. 2018 [16]	Serious	Moderate	Low	Low	Low	Moderate	Moderate	Serious
Konan et al. 2011 [17]	Serious	Moderate	Low	Low	Moderate	Moderate	Moderate	Serious
Kuschnaroff Contreras et al. 2010 [18]	Serious	Serious	Low	Low	Moderate	Moderate	Moderate	Serious
Lee et al. 2013 [19]	Serious	Moderate	Low	Low	Low	Moderate	Moderate	Serious
Nossa et al. 2014 [21]	Moderate	Moderate	Low	Low	Low	Moderate	Moderate	Moderate
Park MS et al. 2014 [24]	Serious	Moderate	Low	Low	Moderate	Moderate	Moderate	Serious
Philippon et al. 2020 [25]	Serious	Moderate	Low	Low	Low	Moderate	Moderate	Serious
Schüttler KF et al. 2018 [30]	Moderate	Moderate	Low	Low	Moderate	Moderate	Moderate	Moderate
Smith KM et al. 2017 [32]	Serious	Moderate	Low	Low	Low	Moderate	Moderate	Serious
Tassinari E et al. 2025 [36]	Serious	Moderate	Low	Low	Low	Moderate	Moderate	Serious
Vilchez F et al. 2010 [37]	Serious	Serious	Low	Low	Moderate	Serious	Serious	Serious
You et al. [39]	Moderate	Low	Low	Low	Low	Low	Low	Moderate

Abbreviation: ROBINS‐I, Risk Of Bias In Non‐randomized Studies—of Interventions.

### GRADE assessment of certainty of evidence

Certainty of evidence was low to very low across outcomes (Table 3), mainly due to risk of bias and inconsistency.

**Table 3:** GRADE assessment.

Outcome	No. of studies	Study design	Key limitations	Consistency	Certainty of evidence (GRADE)
Operative time	7	Observational (cohort/case series)	Serious risk of bias due to confounding and selection; heterogeneity in learning‐curve definitions	Inconsistent	Low
Traction time	7	Observational (cohort/case series)	Serious risk of bias; imprecision and heterogeneity across studies	Inconsistent	Very low
Functional MCID	9	Observational (cohort studies)	Serious risk of bias; indirectness due to varying MCID definitions	Inconsistent	Low
mHHS	6	Observational (cohort/case series)	Serious risk of bias; heterogeneity in reporting and follow‐up	Inconsistent	Low
HOS‐SSS	3	Observational (cohort studies)	Serious risk of bias; imprecision due to small number of studies	Inconsistent	Very low
NAHS	4	Observational (cohort/case series)	Serious risk of bias; heterogeneity in outcome reporting	Inconsistent	Low
VAS Pain	2	Observational (cohort studies)	Very serious imprecision due to few studies; risk of bias	Inconsistent	Very low
Overall complications	6	Observational (cohort/case series)	Serious risk of bias; inconsistent complication definitions	Moderately consistent	Low
Conversion to THA	5	Observational (cohort/registry‐based)	Serious risk of bias; rare events and imprecision	Inconsistent	Very low
Revision arthroscopy	3	Observational (cohort/case series)	Serious risk of bias; imprecision due to low event rates	Inconsistent	Very low

Abbreviations: GRADE, Grading of Recommendations Assessment, Development and Evaluation; HOS‐SSS, Hip Outcome Score—Sports‐Specific Subscale; MCID, minimal clinically important difference; mHHS, modified Harris Hip Score; NAHS, Non‐Arthritic Hip Score; THA, total hip arthroplasty; VAS, Visual Analogue Scale.

### Publication bias

Funnel plots showed no consistent evidence of major publication bias (Figures [Link], [Link], [Link], [Link], [Link], [Link], [Link], [Link], [Link], [Link]). Mild asymmetry for operative and traction time suggested small‐study effects. For several outcomes, interpretation was limited by the small number of studies.

### Meta‐analysis

#### Operative time

Across seven studies [5, 15, 19, 30, 36, 37, 39] reporting operative time, mean differences between late and early cases ranged from –60.0 [37] to –8.6 min [39]. All but one study demonstrated shorter operative times in late cases.

**Figure 2:**
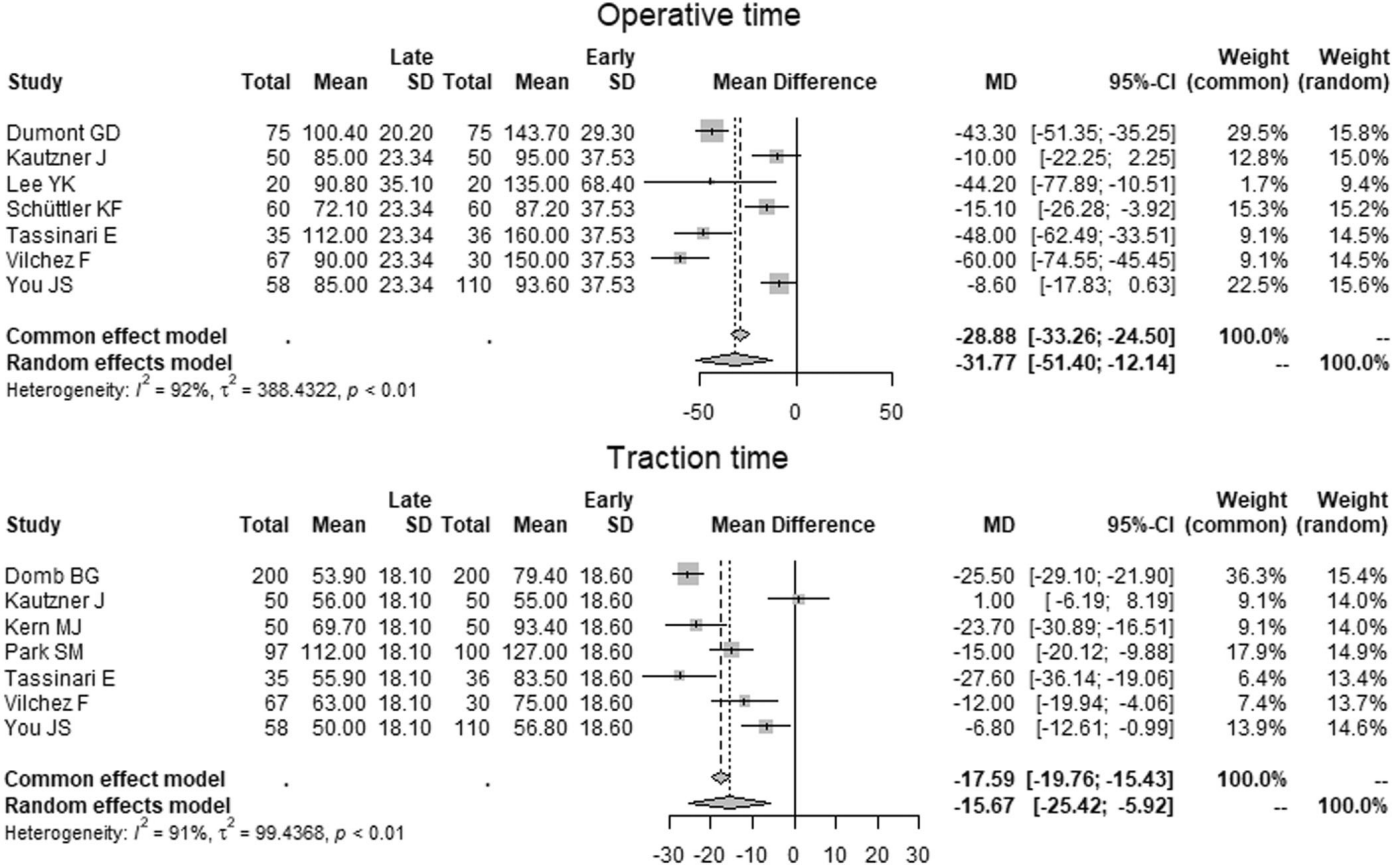
Forest plot of operative time (min.) and traction time (min.). CI, confidence interval; MD, mean difference; SD, standard deviation.

The pooled estimate under the random‐effects model showed a statistically significant reduction in operative time of –31.77 min (95% confidence interval [CI] –51.40 to –12.14). Statistical heterogeneity was high (*I* ^2^ = 92%, *τ* ^2 ^= 388.43, *p* < 0.01) (Figure 2, Table 4).

**Table 4:** Summary of pooled outcomes from the meta‐analysis.

Parameter	Primary studies (*N*)	Hips (*N*)	Treatment effect	CIs	*p* value	*I* ^2^	*τ* ^2^	Egger bias	Egger *p* value
Operative time	7	746	−31.77	−51.4; −12.14	0.0075**	0.92	388.43	−1.91	0.6721
Traction time	7	1133	−15.67	−25.42; −5.92	0.0077**	0.91	99.44	4.53	0.3336
Functional MCID	9	1257	0.58	0.17; 0.99	0.0119*	0.68	0.2	1.33	0.2965
mHHS	6	1017	2.78	0.16; 5.41	0.0416*	0	1.84	−0.22	0.7764
HOS‐SSS	3	720	2.54	−7.41; 12.48	0.387	0	3.76	−2.59	0.3731
NAHS	4	640	8.41	1.99; 14.83	0.0251*	0.47	7.15	2.32	0.25
VAS Pain	2	460	−0.83	−3.97; 2.3	0.1831	0	0.01	N/A	N/A
Complication	6	1078	0.42	0.12; 1.51	0.1416	0.81	1.17	2.53	0.3575
THA conversion	4	440	0.1	0.01; 0.98	0.0487*	0.49	1.16	−1.46	0.5696
Revision	3	340	0.31	0.01; 10.95	0.2919	0.44	1.1	−2.18	0.2633

*Note*: *significant; **more significant.

Abbreviations: CI, confidence interval; HOS‐SSS, Hip Outcome Score—Sports‐Specific Subscale; MCID, minimal clinically important difference; mHHS, modified Harris Hip Score; N/A, not applicable; NAHS, Non‐Arthritic Hip Score; THA, total hip arthroplasty; VAS, Visual Analogue Scale.

#### Traction time

Across seven studies [4, 15, 16, 24, 36, 37, 39] reporting traction time, mean differences between late and early cases ranged from –27.6 [36] to 1.0 min [15]. Most studies favoured shorter traction times in late cases.

The pooled random‐effects estimate demonstrated a statistically significant reduction in traction time of –15.67 min (95% CI –25.42 to –5.92). Heterogeneity was substantial (*I* ^2^ = 91%, *τ* ^2^ = 99.44, *p* < 0.01) (Figure 2, Table 4).

### Functional MCID

Across nine studies [2, 4, 7, 15, 17, 19, 24, 25, 30] reporting functional MCID, mean differences between late and early cases ranged from 0.0 [25] to 1.5 [15]. Most studies demonstrated higher rates of achieving MCID in late cases.

**Figure 3:**
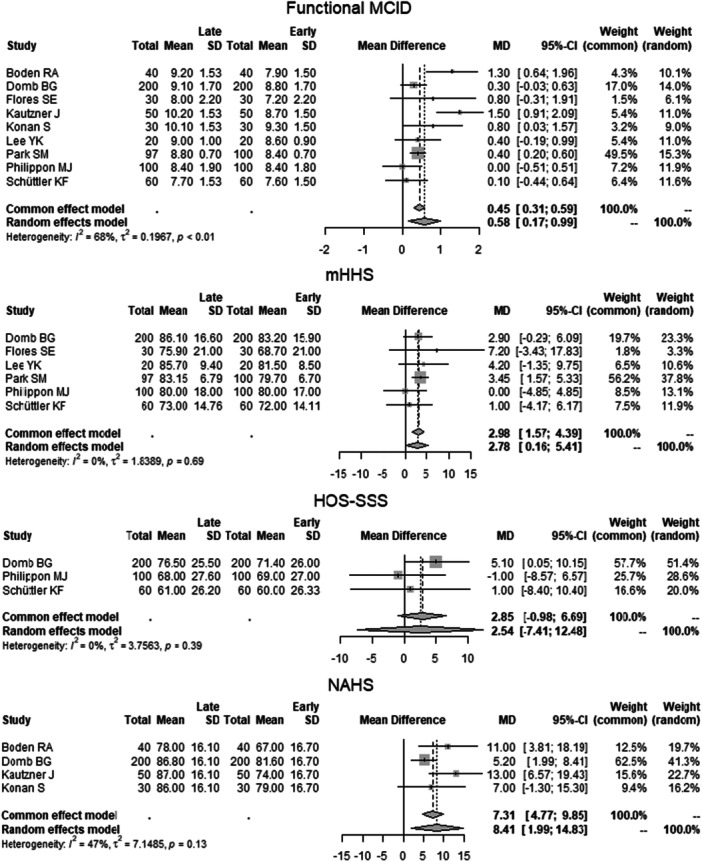
Forest plot of functional MCID, mHHS, HOS‐SSS and NAHS. CI, confidence interval; HOS‐SSS, Hip Outcome Score—Sports‐Specific Subscale; MCID, minimal clinically important difference; MD, mean difference; mHHS, modified Harris Hip Score; NAHS, Non‐Arthritic Hip Score; SD, standard deviation.

The pooled random‐effects estimate demonstrated a statistically significant difference favouring late cases (MD 0.58; 95% CI 0.17 to 0.99). Heterogeneity was substantial (*I* ^2^ = 68%, *τ* ^2^ = 0.20, *p* < 0.01) (Figure 3, Table 4).

### mHHS

Across six studies [4, 7, 19, 24, 25, 30] reporting mHHS, mean differences between late and early cases ranged from 0.0 [25] to 7.2 points [7]. The majority of studies reported higher postoperative scores in late cases.

The pooled random‐effects estimate showed a statistically significant improvement in mHHS for late cases (MD 2.78; 95% CI 0.16 to 5.41). No relevant heterogeneity was observed (*I* ^2^ = 0%, *τ* ^2^ = 1.84, *p* = 0.69) (Figure 3, Table 4).

### HOS‐SSS

Across three studies [4, 25, 30] reporting HOS‐SSS, individual study effects were small and inconsistent.

The pooled random‐effects estimate did not demonstrate a statistically significant difference between late and early cases (MD 2.54; 95% CI –7.41 to 12.48). No statistical heterogeneity was observed (*I* ^2^ = 0%, *τ* ^2^ = 3.76, *p* = 0.39) (Figure 3, Table 4).

### NAHS

Across four studies [2, 4, 15, 17] reporting NAHS, all studies demonstrated higher postoperative scores in late cases, with mean differences ranging from 5.2 to 13.0 points.

The pooled random‐effects estimate demonstrated a statistically significant improvement in NAHS favouring late cases (MD 8.41; 95% CI 1.99 to 14.83). Moderate heterogeneity was observed (*I* ^2^ = 47%, *τ* ^2^ = 7.15, *p* = 0.13) (Figure 3, Table 4).

### Complications

Across six studies [3, 15, 16, 18, 21, 24] reporting overall complications, individual odds ratios ranged from 0.09 to 2.15. Most studies demonstrated lower complication rates in late cases compared with early cases.

**Figure 4:**
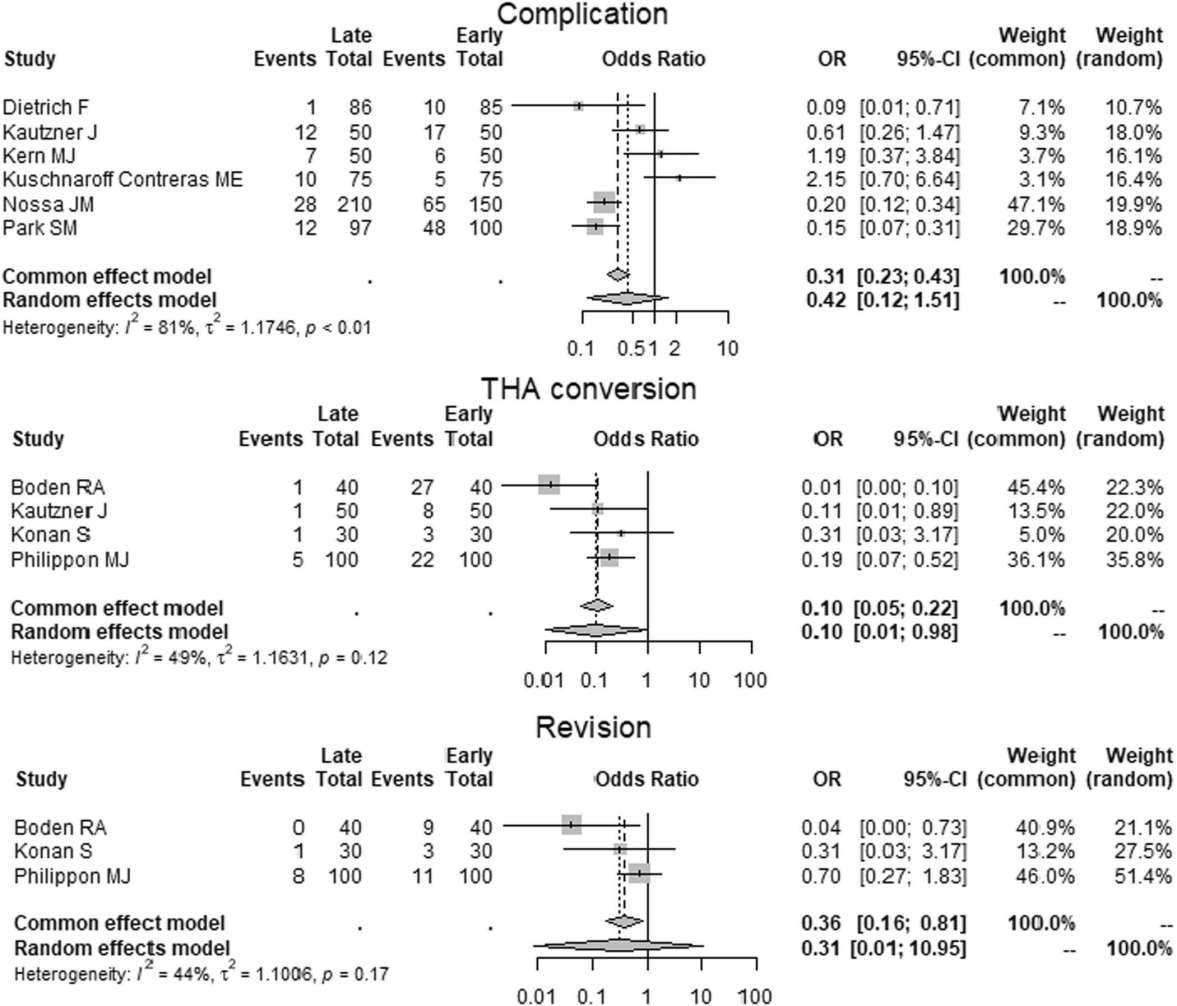
Forest plot of complication, THA conversion and revision rates. CI, confidence interval; OR, odds ratio; THA, total hip arthroplasty.

The pooled random‐effects model did not demonstrate a statistically significant difference in overall complication rates between late and early cases (OR 0.42; 95% CI 0.12 to 1.51). Statistical heterogeneity was substantial (*I* ^2^ = 81%, *τ* ^2^ = 1.17, *p* < 0.01) (Figure 4, Table 4).

### Total hip arthroplasty (THA) conversion

Across four studies [2, 15, 17, 25] reporting conversion to THA, odds ratios consistently favoured late cases, ranging from 0.01 to 0.31.

The pooled random‐effects estimate demonstrated a statistically significant reduction in the risk of conversion to THA for late cases (OR 0.10; 95% CI 0.01 to 0.98). Moderate heterogeneity was observed (*I* ^2^ = 49%, *τ* ^2^ = 1.16, *p* = 0.12) (Figure 4, Table 4).

### Revision

Across three studies [2, 17, 25] reporting revision surgery, individual odds ratios ranged from 0.04 to 0.70, with all studies favouring late cases.

The pooled random‐effects estimate did not demonstrate a statistically significant difference in revision rates between late and early cases (OR 0.31; 95% CI 0.01 to 10.95). Heterogeneity was moderate (*I* ^2^ = 44%, *τ* ^2^ = 1.10, *p* = 0.17) (Figure 4, Table 4).

## DISCUSSION

The main finding of this meta‐analysis is that increasing surgeon experience in HAS is associated with substantial improvements in operative efficiency, reflected by significantly reduced operative and traction times and modest gains in functional outcomes. In contrast, complication and revision rates remained unchanged, whereas conversion to THA was lower in late cases.

### Operative efficiency and learning‐curve dynamics

The most consistent findings relate to operative efficiency, with reductions of approximately 32 min in operative time and 16 min in traction time. These improvements reflect increasing procedural familiarity and optimization of intraoperative workflow, although operative time should be interpreted as a marker of efficiency rather than surgical quality.

Prior studies suggest that initial proficiency may be achieved after 50–100 cases, with higher thresholds for complex procedures [9, 14, 22]. Volume–outcome relationships indicate that learning effects may extend further, with improved outcomes observed at higher cumulative case volumes [20]. Substantial heterogeneity likely reflects variability in case mix, procedural complexity, surgeon experience and institutional factors.

HAS presents specific technical challenges, including constrained joint access and complex pathology management [35], which may limit generalizability. Overall, these findings are consistent with previous reviews [9, 14, 31] and extend them by providing quantitative estimates across outcome domains.

### Functional outcomes

Improvements in PROMs were smaller but consistently favoured late cases. Higher MCID achievement and modest gains in mHHS and NAHS suggest incremental functional benefits with experience. However, effect sizes were limited and approached thresholds of clinical relevance. Functional outcomes are likely influenced more strongly by patient selection and underlying pathology than by surgical experience alone. The absence of differences in HOS‐SSS supports this interpretation. These findings are consistent with recent evidence indicating that improved surgical planning and technical execution may further enhance clinical outcomes in HAS, underscoring the role of experience‐dependent factors in optimizing results [38].

### Safety outcomes

Complication and revision rates were comparable between early and late phases, indicating an acceptable safety profile even during early experience. In contrast, conversion to THA was lower in late cases, although this finding should be interpreted cautiously, given limited data and low certainty of evidence. This effect may reflect improved patient selection and decision‐making rather than technical factors alone.

### Publication bias

No consistent evidence of substantial publication bias was observed. Mild asymmetry for operative and traction time likely reflects small‐study effects. Interpretation was limited for several outcomes due to the small number of studies.

### Methodological limitations

All included studies were non‐randomized and subject to confounding and selection bias. Substantial heterogeneity and non‐standardized definitions of the learning curve limit comparability. Additional uncertainty arises from outcome harmonization, graphical data extraction and MCID‐based normalization. Radiographic outcomes were inconsistently reported and could not be analysed. Overall certainty of evidence was low to very low, and findings should be interpreted as indicative rather than causal.

### Clinical implications

From a clinical perspective, these findings suggest that HAS can be performed safely during the early learning phase under appropriate supervision, while continued experience leads to substantial gains in operative efficiency. This supports structured training pathways, careful case selection during early practice and more informed patient counselling regarding expected outcomes and surgeon experience. Future research should focus on modifiable factors influencing learning, including simulation‐based training and standardized surgical pathways [1]. Prospective multicenter studies may further define experience thresholds associated with optimal outcomes.

## CONCLUSION

Advancing surgical experience in HAS is associated with improved operative efficiency and modest functional gains, while safety outcomes remain largely unchanged. The reduced rate of THA conversion in late cases should be interpreted cautiously, given the observational nature and heterogeneity of the data. Overall, these findings support structured learning while highlighting the need for higher‐quality evidence.

## AUTHOR CONTRIBUTIONS

Nikolai Ramadanov and Maximilian Heinz performed the literature search, the data extraction and the risk of bias assessment. Robert Hable and Nikolai Ramadanov conducted the statistical calculations. Nikolai Ramadanov and Robert Hable created all figures and tables. Nikolai Ramadanov wrote the manuscript. Ingo J. Banke and Roland Becker supervised the work.

## CONFLICT OF INTEREST STATEMENT

The authors declare no conflicts of interest.

## ETHICS STATEMENT

The authors have nothing to report.

## Supporting information


Supporting File 1

Supporting File 2

Supporting File 3

Supporting File 4

Supporting File 5

Supporting File 6

Supporting File 7

Supporting File 8

Supporting File 9

Supporting File 10

Supporting File 11

## Data Availability

Data are available from the corresponding author upon reasonable request.

## References

[1] Arevalo A , Keller R , Szukics P , Olsen C , Arevalo I , Yagnik G , et al. Variation in reported learning outcomes and measurement instruments in hip arthroscopy simulation training: a systematic review. Arthroscopy. 2024;40:176–186. 37355192 10.1016/j.arthro.2023.06.019

[2] Boden RA , Wall AP , Fehily MJ . Results of the learning curve for interventional hip arthroscopy: a prospective study. Acta Orthop Belg. 2014;80:39–44. 24873083

[3] Dietrich F , Ries C , Eiermann C , Miehlke W , Sobau C . Complications in hip arthroscopy: necessity of supervision during the learning curve. Knee Surg Sports Traumatol Arthrosc. 2014;22:953–958. 24519620 10.1007/s00167-014-2893-9

[4] Domb BG , Chen SL , Shapira J , Maldonado DR , Lall AC , Rosinsky PJ . The evolution of hip arthroscopy: what has changed since 2008—a single surgeon's experience. Arthroscopy. 2020;36:761–772. 31919020 10.1016/j.arthro.2019.10.009

[5] Dumont GD , Cohn RM , Gross MM , Menge TJ , Battle NC , Thier ZT . The learning curve in hip arthroscopy: effect on surgical times in a single‐surgeon cohort. Arthroscopy. 2020;36:1293–1298. 31805387 10.1016/j.arthro.2019.11.121

[6] Egger M , Smith GD , Schneider M , Minder C . Bias in meta‐analysis detected by a simple, graphical test. BMJ. 1997;315:629–634. 9310563 10.1136/bmj.315.7109.629 PMC2127453

[7] Flores SE , Borak KR , Zhang AL . Hip arthroscopic surgery for femoroacetabular impingement: a prospective analysis of the relationship between surgeon experience and patient outcomes. Orthop J Sports Med. 2018;6:2325967118755048. 29468172 10.1177/2325967118755048 PMC5815420

[8] Frank RM , Erickson B , Frank JM , Bush‐Joseph CA , Bach Jr. BR , Cole, BJ , et al. Utility of modern arthroscopic simulator training models. Arthroscopy. 2013;29:2220–2233. 10.1016/j.arthro.2013.09.084 24290789

[9] Go CC , Kyin C , Maldonado DR , Domb BG . Surgeon experience in hip arthroscopy affects surgical time, complication rate, and reoperation rate: a systematic review on the learning curve. Arthroscopy. 2020;36:3092–3105. 32679291 10.1016/j.arthro.2020.06.033

[10] Gupta A , Redmond JM , Hammarstedt JE , Schwindel L , Domb BG . Safety measures in hip arthroscopy and their efficacy in minimizing complications: a systematic review of the evidence. Arthroscopy. 2014;30:1342–1348. 25017649 10.1016/j.arthro.2014.04.103

[11] Guyatt GH , Oxman AD , Vist GE , Kunz R , Falck‐Ytter Y , Alonso‐Coello P , et al. GRADE: an emerging consensus on rating quality of evidence and strength of recommendations. BMJ. 2008;336:924–926. 18436948 10.1136/bmj.39489.470347.AD PMC2335261

[12] Guyot P , Ades A , Ouwens MJ , Welton NJ . Enhanced secondary analysis of survival data: reconstructing the data from published Kaplan–Meier survival curves. BMC Med Res Methodol. 2012;12:9. 22297116 10.1186/1471-2288-12-9 PMC3313891

[13] Haipeng L , Ji L , Juanli Z , Lijun S , Yujie L , Zhongli L , et al. Portal setup: the key point in the learning curve for hip arthroscopy technique. Orthop Surg. 2021;13:1781–1786. 34664419 10.1111/os.13035 PMC8523757

[14] Hoppe DJ , de Sa D , Simunovic N , Bhandari M , Safran MR , Larson CM , et al. The learning curve for hip arthroscopy: a systematic review. Arthroscopy. 2014;30:389–397. 24461140 10.1016/j.arthro.2013.11.012

[15] Kautzner J , Zeman P , Stančák A , Havlas V . Hip arthroscopy learning curve: a prospective single‐surgeon study. Int Orthop. 2018;42:777–782. 29046931 10.1007/s00264-017-3666-0

[16] Kern MJ , Murray RS , Sherman TI , Postma WF . Incidence of nerve injury after hip arthroscopy. J Am Acad Orthop Surg. 2018;26:773–778. 30180092 10.5435/JAAOS-D-17-00230

[17] Konan S , Rhee SJ , Haddad FS . Hip arthroscopy: analysis of a single surgeon's learning experience. J Bone Jt Surg. 2011;93(Suppl 2):52–56. 10.2106/JBJS.J.01587 21543689

[18] Kuschnaroff Contreras ME , Hoffmann RB , de Araújo LC , Dani WS , José Berral F . Complications in hip arthroscopy. Rev Bras Ortop. 2015;45:61–66. 27022521 10.1016/S2255-4971(15)30218-4 PMC4799139

[19] Lee YK , Ha YC , Hwang DS , Koo KH . Learning curve of basic hip arthroscopy technique: CUSUM analysis. Knee Surg Sports Traumatol Arthrosc. 2013;21:1940–1944. 23073816 10.1007/s00167-012-2241-x

[20] Mehta N , Chamberlin P , Marx RG , Hidaka C , Ge Y , Nawabi DH , et al. Defining the learning curve for hip arthroscopy: a threshold analysis of the volume‐outcomes relationship. Am J Sports Med. 2018;46:1284–1293. 29337602 10.1177/0363546517749219

[21] Nossa JM , Aguilera B , Márquez W , Aranzazu A , Alzate R , Rueda G , et al. Factors associated with hip arthroscopy complications in the treatment of femoroacetabular impingement. Curr Orthop Pract. 2014;25:362–366.

[22] Oduguwa E , Price A , Awal A , Kraeutler M . EP238 Assessing the learning curve in hip arthroscopy: a prospective case series analysis of operative efficiency over time. J Hip Preserv Surg. 2025;12(Suppl 2):ii104.

[23] Page MJ , McKenzie JE , Bossuyt PM , Boutron I , Hoffmann TC , Mulrow CD , et al. The PRISMA 2020 statement: an updated guideline for reporting systematic reviews. BMJ. 2021;372:n71. 33782057 10.1136/bmj.n71 PMC8005924

[24] Park MS , Yoon SJ , Kim YJ , Chung WC . Hip arthroscopy for femoroacetabular impingement: the changing nature and severity of associated complications over time. Arthroscopy. 2014;30:957–963. 24835839 10.1016/j.arthro.2014.03.017

[25] Philippon MJ , Utsunomiya H , Locks R , Briggs KK . First 100 segmental labral reconstructions compared to the most recent 100: the role of surgeon experience in decreasing conversion to total hip arthroplasty. Knee Surg Sports Traumatol Arthrosc. 2020;28:2295–2301. 31511918 10.1007/s00167-019-05692-z

[26] Ramadanov N . MCID normalization: a methodological framework for harmonizing heterogeneous PROMs in hip arthroscopy research. J Exp Orthop. 2025;12:e70568. 41245715 10.1002/jeo2.70568 PMC12619535

[27] Ramadanov N , Heinz M , Voss M , Hable R , Becker R , Ahmad SS . Learning curve in periacetabular osteotomy for developmental dysplasia of the hip: a systematic review and meta‐analysis. Bone Joint Open. 2026;7(4):574–583. 42015757 10.1302/2633-1462.74.BJO-2025-0371.R1 PMC13100689

[28] Ramadanov N , Voss M , Diallo RM , Lettner J , Hakam HT , Prill R , et al. Do meta‐analyses of total hip arthroplasty produce reliable results? A systematic review and meta‐epidemiological study of statistical methods. Orthop Surg. 2025;17:1936–1955. 40425483 10.1111/os.70077 PMC12214412

[29] Rohatgi A. WebPlotDigitizer, Version 4.6. https://automeris.io/WebPlotDigitizer (2022).

[30] Schüttler KF , Schramm R , El‐Zayat BF , Schofer MD , Efe T , Heyse TJ . The effect of surgeon's learning curve: complications and outcome after hip arthroscopy. Arch Orthop Trauma Surg. 2018;138:1415–1421. 29802454 10.1007/s00402-018-2960-7

[31] Shultz C , Levine N , Curtis W , Christian RA , Hendren S , Lau BC . A systematic review of learning curves in orthopaedic sports surgery. Iowa Orthop J. 2025;45:161–178. 40606710 PMC12212312

[32] Smith KM , Duplantier NL , Crump KH , Delgado DA , Sullivan SL , McCulloch PC , et al. Fluoroscopy learning curve in hip arthroscopy‐a single surgeon's experience. Arthroscopy. 2017;33:1804–1809. 28969816 10.1016/j.arthro.2017.03.026

[33] Souza BGS , Dani WS , Honda EK , Ricioli Jr. W , Guimarães, RP , Guimarães RP , et al. Do complications in hip arthroscopy change with experience? Arthroscopy. 2010;26:1053–1057. 20678702 10.1016/j.arthro.2009.12.021

[34] Sterne JA , Hernán MA , Reeves BC , Savović J , Berkman ND , Viswanathan M , et al. ROBINS‐I: a tool for assessing risk of bias in non‐randomised studies of interventions. BMJ. 2016;355:i4919. 27733354 10.1136/bmj.i4919 PMC5062054

[35] Suarez‐Ahedo C , Camacho‐Galindo J , López‐Reyes A , Martinez‐Gómez LE , Pineda C , Domb BG . A comprehensive review of hip arthroscopy techniques and outcomes. SAGE Open Med. 2024;12:20503121231222212. 38249944 10.1177/20503121231222212 PMC10798066

[36] Tassinari E , Caternicchia F , Rosa MD , Castagnini F , Angeletti E , Fantoni V , et al. Functional outcome improvement and surgical time reduction in a single‐surgeon consecutive case series of hip arthroscopy for femoroacetabular impingement: a minimum 5 years follow‐up study. J Exp Orthop. 2025;12:e70022. 39845699 10.1002/jeo2.70022 PMC11751622

[37] Vilchez F , Erquicia J , Tey M . [Learning curve of arthroscopic hip surgery]. Acta Ortop Mex. 2010;24:177–181. 20836373

[38] Zheng F , Daofeng W , Guanyang S , Yue L , Xuesong W . Quantitative evaluation and preoperative planning using three‐dimensional CT profiles improves the accuracy of bony correction under primary hip arthroscopy and clinical outcomes in the setting of cam‐type femoroacetabular impingement syndrome. Orthop Surg. 2025;17(8):2422–2434. 40550743 10.1111/os.70102 PMC12318679

[39] You JS , Flores SE , Friedman JM , Lansdown DA , Zhang AL . The learning curve for hip arthroscopic surgery: a prospective evaluation with 2‐year outcomes in patients with femoroacetabular impingement. Orthop J Sports Med. 2020;8:2325967120959140. 33178877 10.1177/2325967120959140 PMC7592324

