# Supplementary figures and images for: Surgeon experience in hip arthroscopy improves operative efficiency and reduces conversion to total hip arthroplasty: A meta‐analysis
Source: Knee Surg Sports Traumatol Arthrosc. 2026 Jun 26;34(7):2562–75. doi: 10.1002/ksa.70431 (PMC13327485; doi:10.1002/ksa.70431)

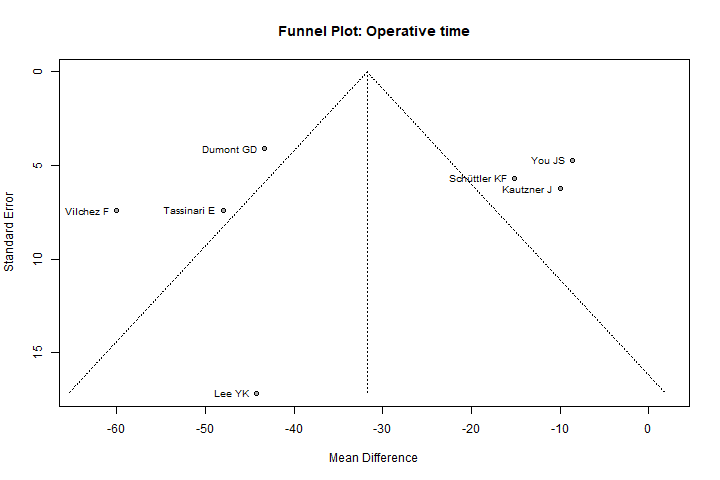

Supplement: Supplementary file 1 — Supporting File 1 [file KSA-34-2562-s007.png]

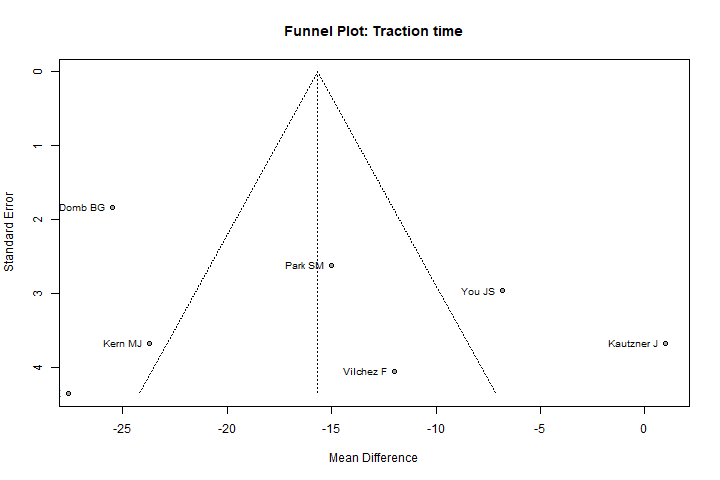

Supplement: Supplementary file 2 — Supporting File 2 [file KSA-34-2562-s009.png]

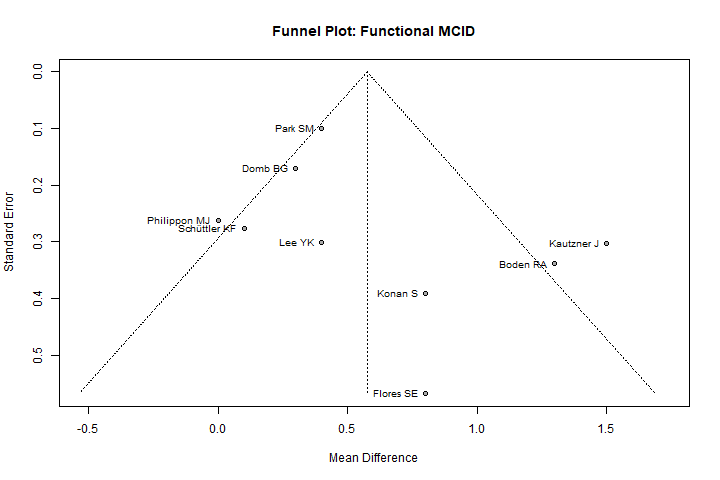

Supplement: Supplementary file 3 — Supporting File 3 [file KSA-34-2562-s001.png]

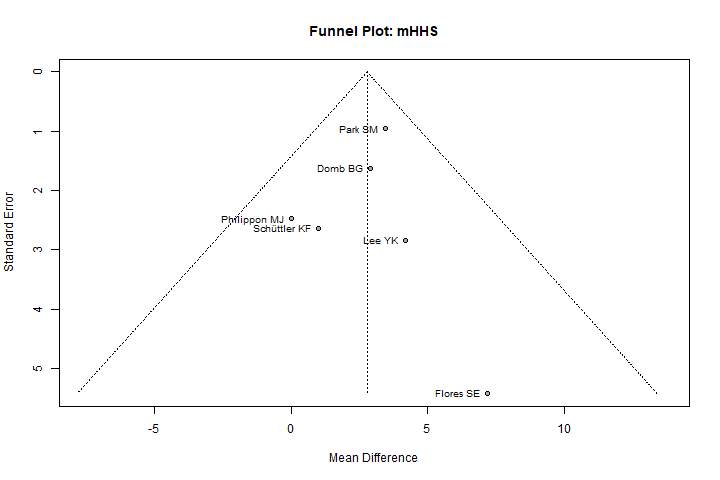

Supplement: Supplementary file 4 — Supporting File 4 [file KSA-34-2562-s004.png]

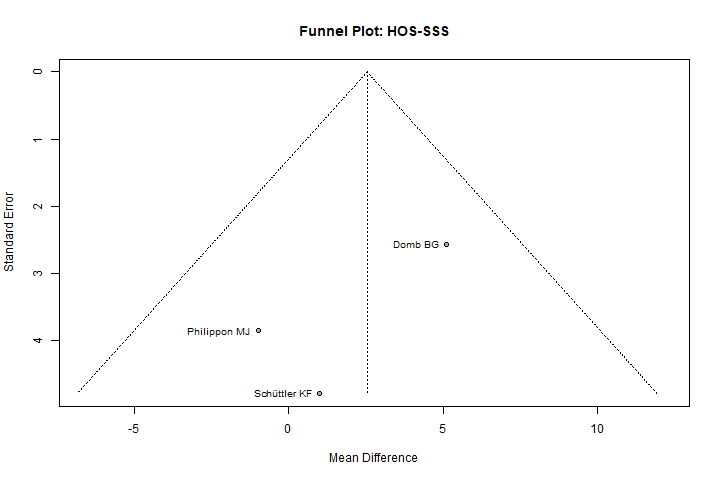

Supplement: Supplementary file 5 — Supporting File 5 [file KSA-34-2562-s002.png]

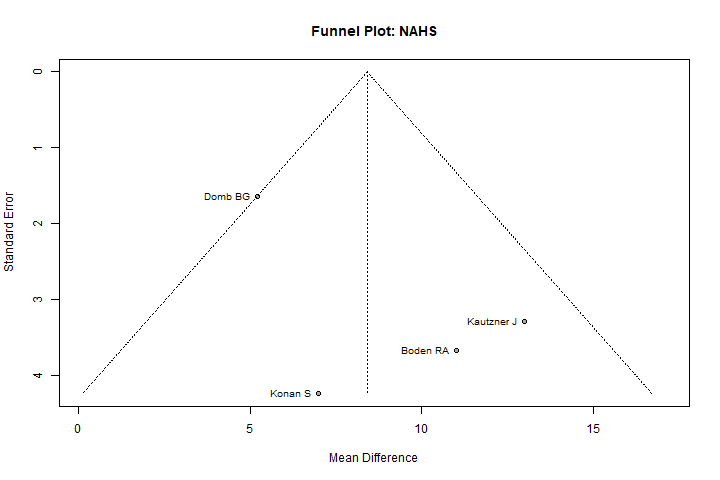

Supplement: Supplementary file 6 — Supporting File 6 [file KSA-34-2562-s003.png]

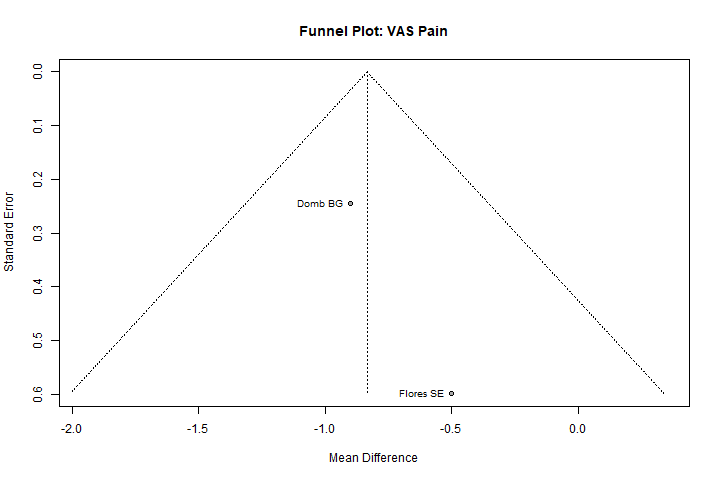

Supplement: Supplementary file 7 — Supporting File 7 [file KSA-34-2562-s010.png]

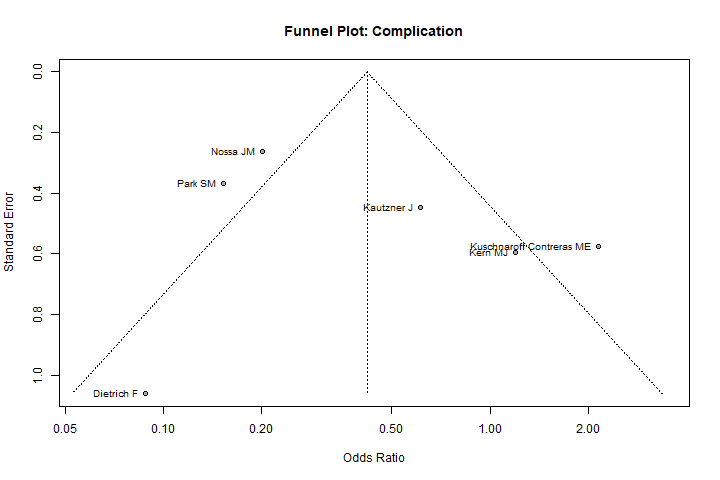

Supplement: Supplementary file 8 — Supporting File 8 [file KSA-34-2562-s006.png]

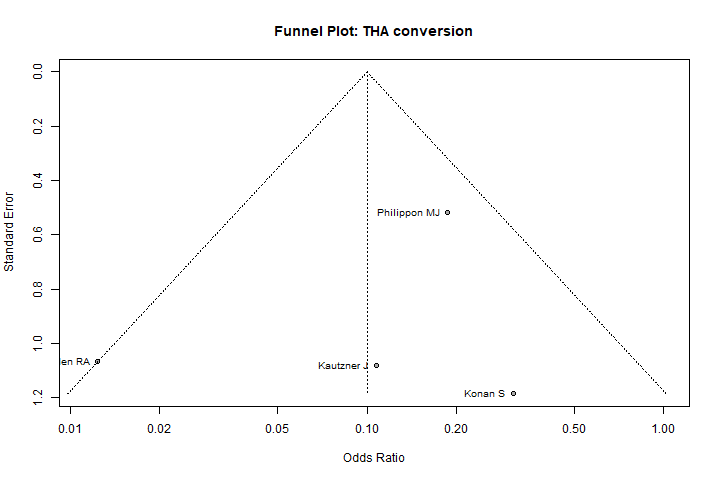

Supplement: Supplementary file 9 — Supporting File 9 [file KSA-34-2562-s008.png]

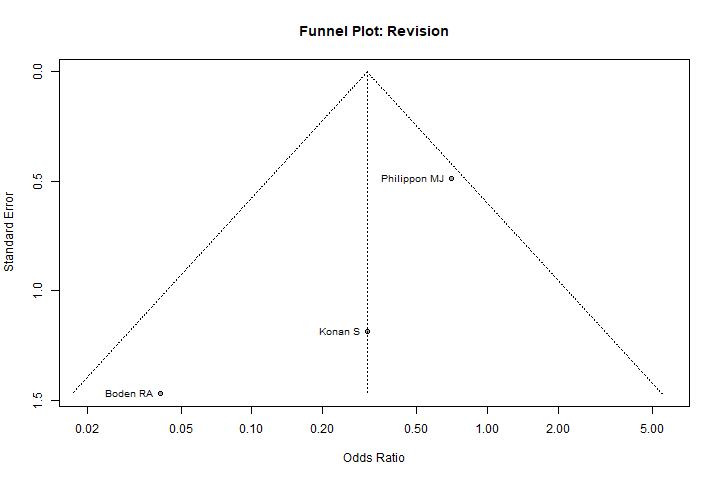

Supplement: Supplementary file 10 — Supporting File 10 [file KSA-34-2562-s011.png]
